# Integrated Expression Profiles of mRNA and miRNA in Polarized Primary Murine Microglia

**DOI:** 10.1371/journal.pone.0079416

**Published:** 2013-11-11

**Authors:** Robert W. Freilich, Maya E. Woodbury, Tsuneya Ikezu

**Affiliations:** 1 Laboratory of Molecular NeuroTherapeutics, Department of Pharmacology and Experimental Therapeutics, Boston University School of Medicine, Boston, Massachusetts, United States of America; 2 Graduate Program in Neuroscience, Boston University, Boston, Massachusetts, United States of America; 3 Department of Neurology and Alzheimer’s Disease Center, Boston University School of Medicine, Boston, Massachusetts, United States of America; Massachusetts General Hospital and Harvard Medical School, United States of America

## Abstract

Neuroinflammation contributes to many neurologic disorders including Alzheimer’s disease, multiple sclerosis, and stroke. Microglia is brain resident myeloid cells and have emerged as a key driver of the neuroinflammatory responses. MicroRNAs (miRNAs) provide a novel layer of gene regulation and play a critical role in regulating the inflammatory response of peripheral macrophages. However, little is known about the miRNA in inflammatory activation of microglia. To elucidate the role that miRNAs have on microglial phenotypes under classical (M1) or alternative (M2) activation under lipopolysaccharide (‘M1’-skewing) and interleukin-4 (‘M2a’-skewing) stimulation conditions, we performed microarray expression profiling and bioinformatics analysis of both mRNA and miRNA using primary cultured murine microglia. miR-689, miR-124, and miR-155 were the most strongly associated miRNAs predicted to mediate pro-inflammatory pathways and M1-like activation phenotype. miR-155, the most strongly up-regulated miRNA, regulates the signal transducer and activator of transcription 3 signaling pathway enabling the late phase response to M1-skewing stimulation. Reduced expression in miR-689 and miR-124 are associated with dis-inhibition of many canonical inflammatory pathways. miR-124, miR-711, miR-145 are the strongly associated miRNAs predicted to mediate anti-inflammatory pathways and M2-like activation phenotype. Reductions in miR-711 and miR-124 may regulate inflammatory signaling pathways and peroxisome proliferator-activated receptor-gamma pathway. miR-145 potentially regulate peripheral monocyte/macrophage differentiation and faciliate the M2-skewing phenotype. Overall, through combined miRNA and mRNA expression profiling and bioinformatics analysis we have identified six miRNAs and their putative roles in M1 and M2-skewing of microglial activation through different signaling pathways.

## Introduction

Inflammation is a dynamic and complex process triggered by tissue insult in any region of the body including the central nervous system (CNS) [1], [2]. The first line of defense is mediated through cells comprising the innate immune system that recognize specific molecular motifs and patterns of foreign invaders [3]. The innate immune system then activates adaptive immunity through antigen presentation to boost the response to a specific target [3]. While the two wings of the immune system function in the CNS as well as in the periphery, there are significant differences in both the sequence of events and the cells recruited to the region affected by the insult in the CNS, primarily due to the presence of blood brain barrier (BBB) [1], [3]. Specifically, the CNS relies much more on innate immunity mediated through microglia, monocytes and macrophages while recruitment of neutrophils and T-cells, which mediate adaptive immunity, is limited [3]–[5].

Peripheral macrophages display plasticity characterized by their phenotypic response to pathogenic stimuli, such as “classical activation” (M1) phenotype characterized by both pro-recruitment and formation of cytotoxic reactive oxygen/nitrogen species. The other known phenotype is “alternative activation” (M2), which exhibit enhanced phagocytosis of apoptotic bodies, anti-inflammatory, and wound healing properties [6]–[11]. The M1 phenotype appears as the default response to lipopolysaccharide (LPS), a prototypical Toll-like receptor 4 (TLR4) ligand, and other pro-inflammatory cytokine stimuli [6], [7]. However, pre-treatment of macrophages with various M2-promoting cytokines can influence the responsiveness to various M2 subtypes such as a pro-cleanup phenotype (M2a) with enhanced phagocytic capability of apoptotic bodies by interleukin-4 (IL-4)/IL-13 stimulation, or wound-healing phenotype (M2c) by IL-10, transforming growth factor-β (TGF-β) or corticosteroid stimulation [6], [7].

In the CNS there is a growing appreciation for the role neuroinflammation contributes in neuropathogenesis of neurodegenerative disorders, such as Alzheimer’s disease (AD), Parkinson’s disease (PD), amyotrophic lateral sclerosis (ALS), multiple sclerosis (MS), stroke, human inmmunodeficiency virus (HIV)-associated dementia and psychiatric illnesses such as depression [12]–[16]. Microglia, originally described by Pio Del Rio-Hortega in 1932, are generally accepted as the resident member of the mononuclear phagocytes in the CNS and have emerged as the driver of the neuroinflammatory response and wound healing [17]. Microglia were initially thought to exist in two states: a “Resting” state with a ramified, branched morphology or an “Activated” state with an amoeboid morphology upon detection of pathogenic stimulation [17]–[19]. Current research suggests that microglia display a similar dynamic phenotypic response to either pathogenic or cytokine stimulation similar to peripheral macrophages, ranging from an M1-like pro-inflammatory activation phenotype to the M2-like alternative activation phenotype [6]–[11], [20], [21]. Colton *et al.*, showed that application of IL-4/IL-13 can equally skew microglia into an M2-like alternatively activated state [8]. Further, Fenn *et al.*, demonstrated enhanced M1 skewing in aged versus adult microglia in an LPS challenge paradigm [20]. Currently, we have limited knowledge in comprehensive characterization of phenotypic activation of microglia.

MicroRNAs (miRNA) are one class of non-coding RNAs that regulate specific gene expression post-transcriptionally and play critical roles in broad biological systems, such as embryogenesis, neural and immune system development, host defense mechanism, and carcinogensis [22], [23]. Recently, the role of miRNAs in regulation of the acute inflammatory response has acquired significant attention with the demonstration that a number of miRNAs are significantly enhanced upon different TLR stimulations in peripheral macrophages, notably miR-155 and miR-146a among others [24]–[26]. Recently, Ponomarev *et al.* demonstrated a key role of miR-124 in microglial differentiation and its ability to suppress inflammatory response of infiltrating macrophages in an experimental autoimmune encephalitis mouse model [27]. However, little is known about the global miRNA response under M1 or M2-skewing conditions in microglia.

The aim of this study was to determine the role that miRNAs have on influencing microgial phenotypes under M1 and M2a skewing conditions. To accomplish this we performed genome-wide gene and miRNA expression analyses using primary cultured murine microglia skewed either to M1 or M2a conditions and compared them to resting microglia (M0). To our knowledge this is the first bioinformatics correlation study of miRNA-mRNA expression profiles obtained from M1 or M2a-skewed primary microglia.

## Materials and Methods

### Primary Culture of Murine Microglia by Percoll gradient centrifugation

All experimental procedures using animals were approved by Institutional Animal Care and Use Committee at Boston University School of Medicine. Microglia were cultured as previously published [28]. Briefly, primary microglia were isolated from P0 or P1 pups from pregnant female CD-1 mice purchased from Charles River Laboratories (Wilmington, MA). Each pup was euthanized for brain isolation. After removal of meninges tissue was minced for 15 minutes with sterile razor blades. The minced tissue was subjected to two serial digestions using 0.25% trypsin (Invitrogen, 25200) and 0.5% deoxyribonuclease I (DNase-I) from bovine pancreas (DN25, Sigma-Aldrich, St. Louis, MO) and trituration by pipetting. Cellular suspension was filtered through a 70-µm pore size nylon cell strainer (352350, BD-Falcon, San Jose, CA), and subjected to density gradient centrifugation for 40 minutes at 200 x *g* using Percoll® (GE Healthcare, 17-0891-01, Fairfield, CT) using a 70% | 37% | 30% | 0% as previously described [29]. Microglia were primarily aggregated +/– ∼1 ml from the 70–37% layer separation and were then washed two times with PBS to remove any residual Percoll™ before counting and plating. Cells were plated at a concentration of 2×10^6^ cells/well of non-coated 6-well plates in Dulbecco’s modified essential media with high glucose (DMEM), 10% fetal bovine serum, 100 U/ml penicillin and10 µ/ml streptomycin (all from Life Technologies Corporation/Invitrogen). The plate was incubated for 1 hour at 37 °C in 5% CO_2,_ and the non-adherent cells (mixed glial cell fraction) were removed from the plate. The adherent cells on the original plate comprise the enriched primary microglia, which is cultured for 5 days in the presence of 10 ng/ml recombinant murine granulocyte macrophage-colony stimulating factor (GM-CSF) (CTK-222, Abazyme, Cambridge, MA) and 10 ng/ml murine macrophage-colony stimulating factor (M-CSF) (CTK-308, Abazyme). We observed >85% enrichment of microglia (Fig. S1A) with minimal neuronal or astrocytic contamination using this approach.

### Primary Microglia Isolation by Magnetic Bead Separation

To enhance the purity of the microglia we performed magnetic bead isolation using CD11b Microbeads (Miltenyi Biotec GmbH, Bergisch Gladbach, Germany) according to the manufacturer’s procedure [30]. Briefly, P0-1 pups from pregnant female CD-1 mice were euthanized and their brains were isolated. A single cell suspension was prepared using neural tissue dissociation protocol (130-093-231, Miltenyi Biotec) and gentleMACS™ Dissociator (130-093-235, Miltenyi Biotec). The microglia in the isolated single cell suspension were captured with CD11b MicroBeads (130-093-634) for 15 min at 4 °C. After washing excess unbound beads by centrifugation of cells, the cells were passed through a single MS column (130-042-201, Miltenyi Biotec), which is preset on the OctoMACS™ magnetic Separator (130-042-108, Miltenyi Biotec) and washed three times to remove any unlabeled cells. The washed MS column was removed from the OctoMACS and the microglia was eluted in PBS. Recovered cells were then cultured for 5 days in the tissue culture with both M-CSF and GM-CSF as described above. We observed >93% purity in recovered microglia (Fig S1B).

### Stimulation and RNA Isolation

The primary cultured microglia were placed in serum free media overnight and then stimulated with 100 ng/ml purified LPS (055:B5 *Escherichia coli*, L2880, Sigma-Aldrich), 20 ng/ml recombinant murine IL-4 (CTK282, Abazyme), or equal volume of PBS for 4 hrs at 37°C with 5% CO_2_. To extract total RNA (combined mRNA and miRNA) samples, the cells are washed once with PBS and then lysed using QIAzol® Lysis Reagent (79306, Qiagen, Germantown, MD) and then isolated using the miRNeasy® Mini Kit (217004, Qiagen) using the automated RNA purification instrument (QIAcube®, 9001292, Qiagen). The isolated RNA is checked for quality by RNA agarose gel electrophoresis, 2100 Bioanalyzer (Agilent Technologies, Santa Clara, CA), having a minimum 260/280 ratio of 1.80 or greater, and then quantified using NanoDrop 1000 (Thermo Scientific, Waltham, MA).

### mRNA gene expression and miRNA expression Profiling Microarrays

For mRNA expression profile, samples were tested on the Affymetrix GeneChip Mouse Exon 1.0 ST Array (900818, Affymetrix, Santa Clara, CA) which targets more than 28,000 annotated genes with over 835,897 distinct probes (approximately 40 probes per gene). For total miRNA expression profiling, samples were tested on the Mouse GeneChip miRNA 2.0 Array (901754, Affymetrix) that interrogates 690 murine pre-miRNAs and 722 murine mature miRNAs, which provides complete coverage of miRBase v15, including both mature and hairpin stem loops. Both mRNA and miRNA expression profiles were performed by Microarray Core facility at the Boston University School of Medicine. The GeneChip data files were deposited to the NCBI Gene Expression Omnibus (GEO) repository with accession numbers of GSE49329 and GSE49330 for the mRNA and miRNA expression profiles, respectively.

### Microarray data analysis

To identify pathways and functions previously ascribed to the differentially expressed mRNAs and miRNAs, expression data files were analyzed using Ingenuity® Pathway Analysis (IPA) tool, a commercially available web-delivered bioinformatics tool (Ingenuity Systems, Redwood City, CA) [31], [32]. Ingenuity functional analysis, canonical pathway analysis, and transcriptional factor analysis were identified by performing IPA core analysis of log2 fold change for LPS or IL-4 stimulated group versus un-stimulated group. For detailed explanation of the IPA core analysis employed please see Materials and Methods S1.

### Identification of miRNA Target datasets

To identify potential miRNA-mRNA interaction profiles we combined publically available miRNA prediction target databases, including miRanda database (http://www.microrna.org) and Ingenuity Systems which combines TargetScan (http://www.targetscan.org) and TarBase (http://diana.cslab.ece.ntua.gr/tarbase/) [33]–[35]. Select differentially regulated miRNAs were correlated with observed gene expression differences and then compared with predicted targets of the miRNA using publically available Gene List Venn Diagram software [36] (http://simbioinf.com/mcbc/applications/genevenn/genevenn.htm).

### Validation of differentially regulated miRNA and mRNA from the genome-wide microarray

Based on the results of the microarray analysis, select miRNA and mRNA targets were verified by real-time reverse transcription Polymerase Chain Reaction (RT-PCR). We used RNA samples (n  =  4) for each stimulation condition from the same RNA used for the miRNA and mRNA microarray testing. mRNA RT-PCR was performed using QuantiTect Reverse transcription kit (205311, Qiagen) for cDNA synthesis, QuantiFast SYBR Green RT-PCR Kit (204154, Qiagen) for real-time PCR, and custom designed primers for target genes (Invitrogen) (see Table S1 for primer sequences for all genes probed). miRNA RT-PCR was performed using miRCURY Locked Nucleic Acid (LNA)™ Universal cDNA Synthesis Kit (203300, Exiqon, Vedbaek, Denmark), miRCURY LNA™ SYBR Green master mix RT-PCR Kit (203450, Exiqon) and specific primers to mmu-miR-155, mmu-miR-124 and mmu-miR-146a (#205080, #204319, and #204688, Exiqon, respectively). All RT-PCR reactions were conducted using the real-time thermal cycler (Mastercycler® ep realplex 4, Eppendorf).

### Statistical Analysis

All data were normally distributed and presented as mean values ± SEM. In the case of single mean comparisons, data was analyzed by Students *t-*test. In the case of multiple mean comparisons one-way analysis of variance and Tukey’s *post hoc* or two-way repeated measures analysis of variance was used (Prism 4.0, Graphpad Software, San Diego, CA, USA). P-values < 0.05 were regarded as significant. Statistical analysis on microarray data was performed with the aid of Boston University School of Medicine’s Microarray Core facility performing Students *t-*tests on miRNA and gene expression arrays, as well as Pearson’s correlation coefficient between select miRNAs and gene expression.

## Results

### Differentially expressed genes in LPS (M1-skewing) and IL-4 (M2a-skewing) stimulated Primary murine microglia

**Figure 1:**
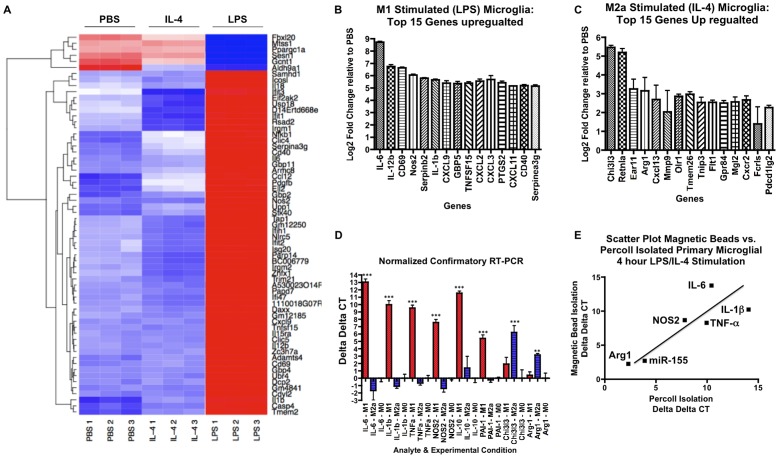
Summary of genome-wide mRNA profiles of M1- or M2a-skewed microglia. (A) Heatmap of top 63 differentially regulated genes among the M1-skewed (LPS), M2a-skewed (IL-4) and M0 resting microglia from mRNA microarray analysis (Moderated FDR q<1×10^−7^). Color scheme: Blue (below average), white (average) and red (above average). 1, 2, and 3 are replicates for each individual microarray of the LPS, IL-4 or PBS-treated conditions. (B-C) Top 15 up-regulated genes in M1-skewed microglia (B) or M2a-skewed microglia (C) (n = 3, p<1×10^−5^ vs. PBS group as determined by Student’s *t*-test). (D) Real-time RT-PCR test of select genes in M0, M1 or M2a-skewed microglia. Canonical M1 markers (IL-6, IL-1β, TNF-α and NOS2), M2b markers (IL-10 and PAI-1) and M2a markers (Chi3l3 and Arg1) were tested. ΔΔCT values were vs. M0 group (n = 4, ** or *** denotes p<0.01 or 0.001, respectively). (E) Scatter plot comparison of real time RT-PCR (or LNA-based RT-PCR for miRNA) ΔΔCT values of select genes and miRNA in microglia isolated by Percoll® density gradient (X-axis) vs. microglia isolated by magnetic beads (Y-axis). Cells were cultured for five days and stimulated with LPS (for IL-6, NOS2, IL-1β, TNF-α, or miR-155 expression) or IL-4 (for Arg1 expression). Pearson’s correlation coefficient: r^2^ = 0.72.

Resting, M1-classical, or M2-alternative skewing of primary murine microglia was achieved by stimulation of serum-starved cells with PBS, LPS, or IL-4 respectively (Fig. S1C). Cells were then lysed and total RNA was isolated, which includes both mRNA and miRNA. The isolated total RNA was subjected to quality examination and samples of high quality RNA were subjected to Affymetrix Mouse Exon 1.0 ST Array for mRNA expression profiling. M1-skewed primary microglia differentially regulated a total of 4275 genes out of total 28,853 genes to a significance of p < 0.0001, where 2477 genes (58%) were upregulated and 1798 genes (42%) were downregulated (Table 1). A differential mRNA expression profile is presented as heat map, highlighting 63 genes that were differentially regulated to a significance determined by a moderated FDR q < 1×10^−7^ as compared against resting microglia (Fig. 1A). Consistent with previous reports in both microglia and macrophages, we found the IL-6 gene to be the most upregulated gene upon LPS stimulation, showing 425-fold increase relative to resting cells (Fig. 1B) [37], [38]. Along with IL-6, the expression of many other canonical M1-marker genes in peripheral macrophages, e.g. IL-12α, NOS2, and IL-1β, are hiγhly enhanced by LPS stimulation of microglia (Fig 1B) [37], [38]. To validate our microarray results we performed mRNA RT-PCR using total RNA isolated from the same samples on select M1-marker genes, and confirmed that expression of IL-6, IL-1β, and NOS2 genes were significantly induced upon LPS stimulation (Fig. 1D). Further, we also demonstrated a significant expression of TNF-α gene (Fig. 1D), which showed 28-fold induction in our mRNA microarray dataset (data not shown). To minimize the potential that these findings included the gene expression profiles of mixed glial cells we performed a second isolation using CD11b magnetic beads and column isolation, as described in Methods. We observed similar induction of in IL-6, TNF-α and IL-1β upon LPS stimulation, which correlated well with the overall tendencies (r^2^ = 0.72) when compared with our initial findings (Fig. 1E and S2A). Further, these findings are consistent with the transcriptional profiles of the M1-skewed peripheral macrophages [39]. Taken together, these data demonstrates classical M1-skewing of microglia by LPS.

**Table 1:** Overall differentially regulated genes (p<0.0001) and miRNAs (p<0.05) by M1 (LPS) and M2a (IL-4) stimulation.

	M1 Stimulation (LPS)			M2a Stimulation (IL-4)		
**mRNA**	4275 (20%)	UP	2477	1606 (7.6%)	Up	905
		Down	1798		Down	701
**miRNA**	47 (6.5%)	UP	12	44 (6.1%)	UP	16
		Down	35		Down	28

We next characterized the gene expression profile of M2-skewed microglia by IL-4 stimulation. A total of 1606 genes were differentially regulated to a significance of p<0.0001, where 905 genes (56%) were up-regulated and 701 genes (44%) were down-regulated (Table 1). We observed the Chitinase-3 Like 3 (CHI3L3) gene, a known M2-marker in peripheral macrophages, as the most up-regulated gene (38.85-fold increase) in IL-4 stimulated microglia (Fig. 1C) [8], [40]. Further, we observed enhanced expression of other M2-markers including RETNLA (FIZZ1) and arginase 1 (ARG1, Fig. 1C) [8], [40]–[42]. To validate the microarray results we performed real-time RT-PCR on select genes and confirmed that gene expression of both CHI3L3 and ARG1 were significantly increased upon IL-4 stimulation (Fig. 1C). We also tested microglia isolated by magnetic bead isolation for IL-4 stimulation, and confirmed similarly enhanced expression of ARG1 (5.8-fold increase by Percoll-isolated microlia and a 4.2-fold increase by magnetic bead isolated microglia, Fig. 1E and S2A). These findings are comparable to the gene transcription profile of M2a-skewed peripheral macrophages [43]. Taken together, these data demonstrate alternative activation of M2-skewed microglia [11], [44].

### Differentially expressed miRNA in LPS and IL-4 stimulated microglia

**Figure 2:**
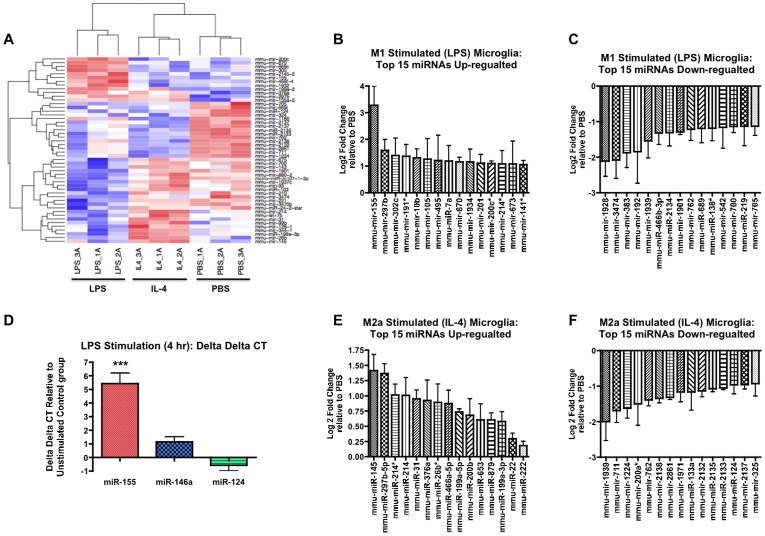
Summary of genome-wide miRNA profiles of M1- or M2a-skewed microglia. (A) Heatmap of top 50 differentially regulated miRNAs (p<0.05 for all miRNAs identified). Color scheme: Blue (below average), white (average) and red (above average). 1A, 2A, and 3A are replicates for each individual microarray of the LPS, IL-4 or PBS treated conditions. (B-C) Top 15 differentially expressed miRNAs up-regulated (B) or down-regulated (C) in M1-skewed microglia (n = 3, p<0.05 vs. PBS group as determined by Student’s *t*-test). (D) LNA-based real-time RT-PCR tests of select miRNAs. ΔΔCT values were calculated relative to PBS group (n = 4, *** denotes p < 0.001 vs. PBS group). (E-F) Top 15 differentially expressed miRNAs up-regulated (E) or down-regulated (F) in M2a-skewed microglia (n = 3, p<0.05 vs. PBS group as determined by Student’s *t*-test).

We next sought to identify miRNAs differentially expressed in the resting, M1- or M2-skewed microglia. The same RNA samples tested for mRNA expression profiling were tested for miRNA microarray expression profiling using the Affymetrix Mouse GeneChip miRNA 2.0 Array. The miRNA expression profile is presented in heat-map by highlighting the top 50 miRNAs (p < 0.05 as determined by Student’s *t*-test vs. resting microglia, Fig. 2A). During LPS stimulation, the expression of 47 out of 722 miRNAs was significantly changed; 12 (26%) miRNAs were significantly increased and 35 (74%) were significantly reduced (Table 1). Interestingly, IL-4-stimulated microglia significantly changed the expression of 44 miRNAs (p<0.05): 16 (36%) miRNAs were increased and 28 (64%) mRNAs were decreased (Table 1).

Under LPS stimulation of microglia, miR-155 was the most significantly up-regulated miRNA (9.69-fold increase vs. PBS control, p<0.01, Fig. 2B). Other significantly increased miRNAs in M1-skewed microglia include: miRs-297b-5p, -302c, -191*, -10b, -105, -495, -7a, -670, -1934, -201, -200c*, -214*, -673, and -141* (Fig. 2B). Further, we also observed significant reduction in the expression of miRs-1928, -3474, -383, -192, -1939, -466b-3p, -2134, -1901, -762, -689, -128*, -542, -700, -219, and -705 (p<0.05) (Fig. 2C). We next performed LNA-based real-time RT-PCR and confirmed that miR-155’s expression is significantly increased upon LPS stimulation (43-fold increase, p < 0.01, Fig. 2D). To validate our results we performed the LNA-based RT-PCR of miR-155 using magnetic bead isolated microglia, and saw a similar increase in miR-155 expression after 4-hour LPS stimulation (Fig. 1E and S2B).

In IL-4 stimulated microglia we observed miR-145 as the most increased miRNA (2.66-fold) along with miR-297b-5p and miR-214 (all greater than 2.0-fold increase, p<0.05, Fig. 2E). Further, there was significant reduction of the expression of miRs-1939, -711, -1224, -200a*, -762, -2138, -2861, -1971, -133a, -2132, - 2135, -2133, -124, -2137, and -325 (p<0.05) (Fig. 2F).

Overall, we have identified miRNAs miR-155 and miR-145 as the most significantly up-regulated miRNAs in the M1 or M2a-skewed microglia, respectively. Further, we also have identified significant down-regulation of miR-689 and miR-711 in the M1 or M2a skewed primary microglia, respectively.

### Systems Biological analysis of differentially expressed genes in LPS (M1-skewing) and IL-4 (M2-skewing) stimulated Primary murine microglia

To characterize how the gene and miRNA expression in different activation states alter microglial functionality, the enrichment pathway analysis was performed using the Ingenuity® Pathway Analysis (IPA) tool [31], [32]. Microglia altered 54 biological functions under the M1-skewing condition. The top 10 up- or down regulated biological functions are identified (Fig. S3A and S3B). Consistent with our expectations, IPA analysis identified the “Inflammatory Response/Immune Response” function as the most significantly up-regulated function enriched with over 280 genes (p<3.59×10^–63^, Fig. S3A). Further, microglia altered 66 biological functions under the M2a-skewing condition; the top 10 up-regulated and down regulated biological functions are identified (Fig. S3C and S3D). IPA analysis also identified the function “Inflammatory Response/Immune Response” as the most significantly up-regulated function enriched with over 84 genes in M2-skewing condition (p<3.70×10^–26^, Fig S5C). In fact, IPA analysis identified that nine out of the top ten functions for both skewing conditions fall in the same categories, leading us to question if these are unique phenotypes (Fig S3A and S3C). To determine if M1-skewing and M2a-skewing actually activated distinct sets of genes, we compared the enriched gene sets from the IPA analysis for both conditions that comprised the “Inflammatory Response/Immune response” function. We found only 33 common genes out of a total of 292 genes (Fig. S3E). From those observations, we concluded that while the top 10 up-regulated functions fall in the same categories between the two phenotypes, each common function comprises mostly distinct genes and ultimately represent a unique phenotype.

**Figure 3:**
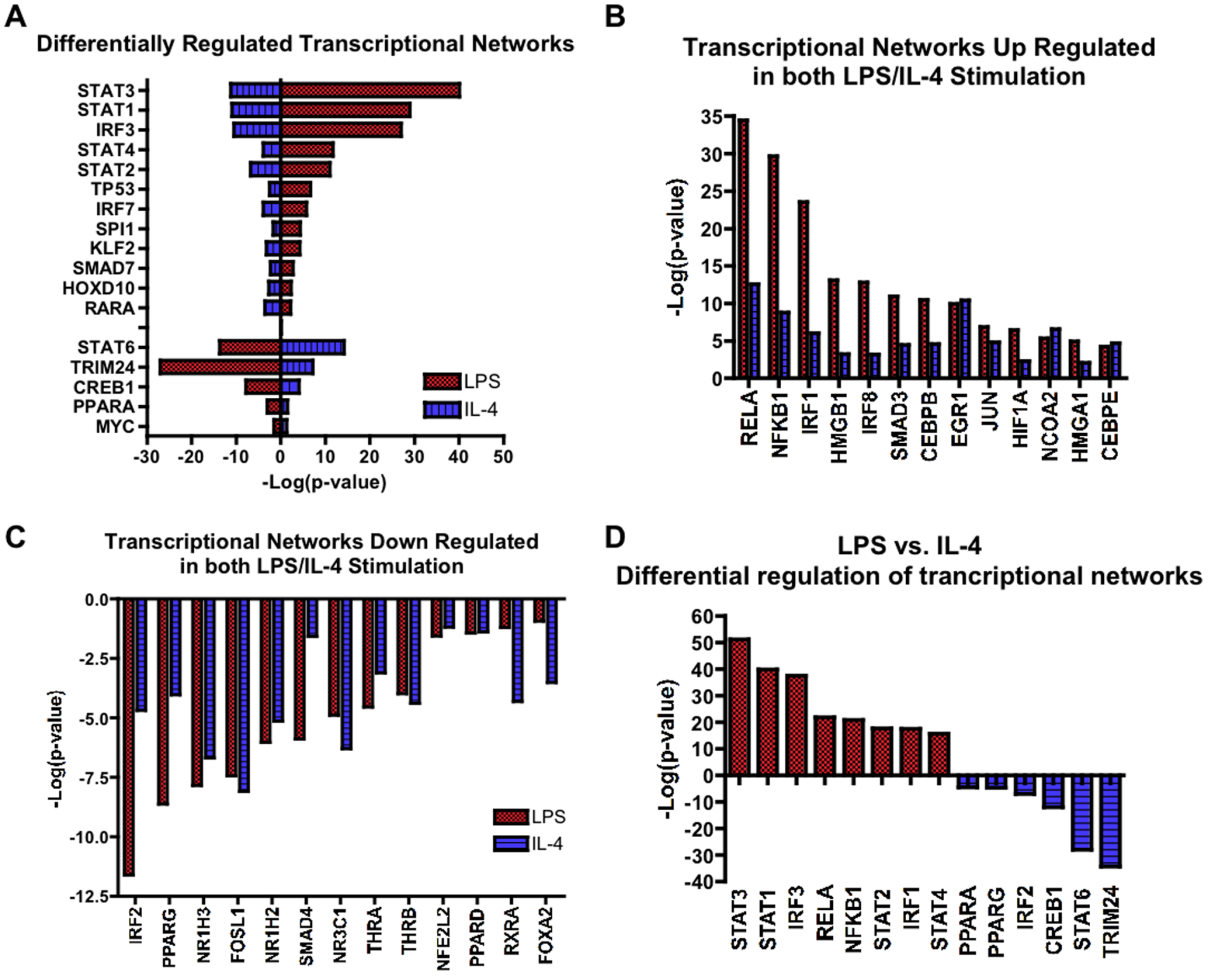
Summary of transcriptional regulatory analysis . (A-D) Comparison of IPA transcriptional network regulation based on enrichment of targets and gene expression changes in transcription factors between M1-skewed (red) and M2a-skewed (blue) microglia. (A) Comparison of differentially regulated transcriptional networks. Negative –Log(p-values) denote down-regulation and positive –Log(p-values) denote up-regulation of the transcriptional network. (B-C) Transcriptional networks that are up-regulated (B) or down-regulated (C) in both M1 and M2a-skewed microglia. (D) A comparison of the most differentially regulated transcriptional networks between M1- and M2a-skewed microglia.

We next looked at the alterations in the transcriptional networks in M1- or M2a- skewed microglia. The IPA tool makes predictions of transcriptional networks based on statistical analysis of how a given condition alters both the transcription factor and its downstream targets (see Materials & Methods S1 for detailed description). LPS stimulation demonstrated many well characterized pro-inflammatory transcription factors as the most significantly “activated” genes, including: NF-κB (NFκB1-RelA), Activating Protein-1 (AP-1), Signal Transducer and Activators of Transcription (STAT 1-4), and Interferon Regulatory Factors (IRF 1, 3, 7 and 8, Fig. 3A-D). Nine other significantly “activated” transcription factors are associated with the pro-inflammatory state of either microglia or macrophages (Fig. 3A). We next characterized the underlying transcriptional regulators in IL-4 stimulated microglia. Consistent with canonical IL-4 signaling, STAT6 was the most significantly “activated” transcription factor (p<6.06×10^–16^, Fig. 3A). Interestingly, there were significant reductions in expression of STAT1, STAT3, STAT4 and IRF3a gene, which all play critical roles in TLR4 signaling (Fig. 3A). Under IL-4 stimulation, we also detected modest but statistically significant up-regulation of many transcription factors that were also seen by LPS stimulation including: NF-κB (NFκB1-RelA), Jun, ERG1, CEBPB, and IRF1 (Fig. 3B). Interestingly, under either LPS or IL-4 stimulation, we commonly observed significant suppression of many nuclear receptor family members, including peroxisome proliferator-activated receptors (PPAR) family, nuclear receptor 1H (NR1H) family, thyroid hormone receptor-β (THRβ) and retinoid x receptor-α (RXRα, Fig. 3C). This is consistent with recent findings that alterations in the nuclear receptor family contribute to microglial activation [45], [46]. However, most importantly we identified genes that are actively suppressed by LPS stimulation and conversely increased by IL-4 stimulation: STAT6, Tripartite motif-containing 24 (TRIM24), cyclic AMP response element-binding-1 (CREB1), IRF2 and PPARα/γ genes (Fig 3D), which may represent key molecules which direct M2-skewing of microglia.

### Target analysis of key miRNA/mRNA interactions identified in LPS-stimulated murine primary microglia

**Figure 4:**
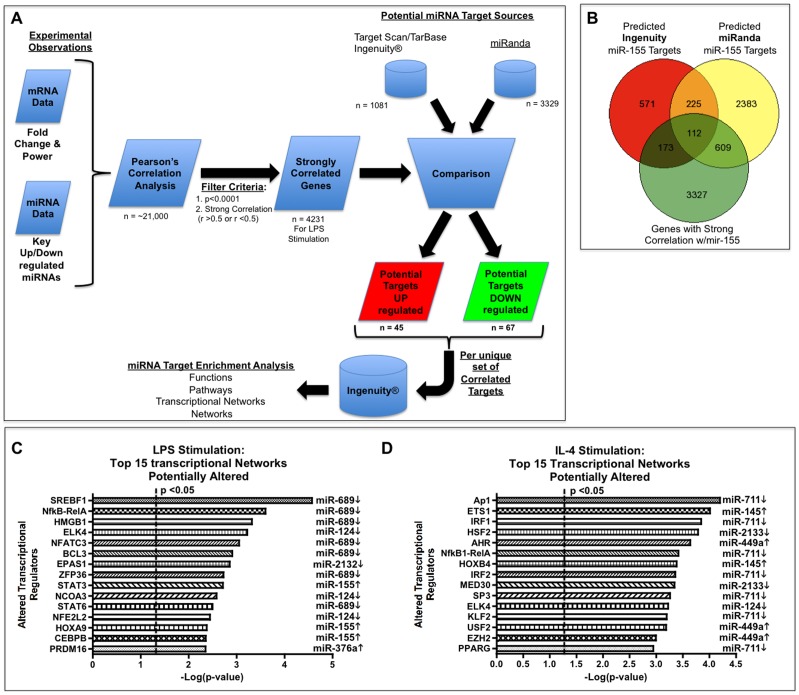
Bioinformatic correlation analysis of miRNA:mRNA interactions in microglia . (A) Method employed for miRNA:mRNA correlation analysis and miRNA potential target enrichment analysis. Briefly, Pearson’s correlation analysis was performed to identify the genes most highly correlated with select miRNA expression profiles. This new gene set was then compared with predicted miRNA targeting gene sets. Common miRNA-correlated target genes were uploaded to Ingenuity® Systems and enrichment analysis was performed to identify targeted functions, targeted pathways, targeted transcriptional networks, and targeted networks. (B) Venn-diagram analysis of representative miRNA:mRNA correlation analysis for miR-155 targets. Predicted targets of miR-155 were derived from public sources (miRanda Database, Ingenuity or TargetScan). miR-155-correlated genes were selected based on two key criteria: a fold change p<0.0001 and strong correlation with miR-155 (r>0.5 or r<–0.5). Venn-diagram shows the intersection gene set of 112 commonly predicted targets that were also strongly correlated with miR-155. (C-D) The top 15 altered transcriptional networks and the corresponding miRNA are presented. IPA-based enrichment analysis was performed on intersected genes for each miRNA to identify key transcriptional networks in the M1-skewed (C) or M2a-skewed microglia (D). Identified transcriptional networks were pooled together from all miRNA altered in the M1- (C) or M2-skewing condition (D) and then sorted by -Log(p-value). Dotted line denotes p<0.05, corresponding to –Log(p-value) > 1.30, as statistical threshold.

To better identify potential miRNA-mRNA interactions that regulate transitions between the different microglia phenotypes, we performed a correlation analysis between select miRNAs and the mRNA expression data sets. To further identify potential miRNA-mRNA interactomes, we next compared the correlated gene sets to potential gene targets of specific miRNAs, identified through use of commercially available target databases, e.g. miRanda, TargetScan (ver 6.0) and TarBase (Fig. 4A) [33]–[35]. We applied this approach to 22 miRNAs representing the most up- or down-regulated miRNAs under M1-skewing conditions (Table S2). On average we observed an 18% overlap between significantly correlated and differentially regulated gene sets when compared our gene expression database against the miRanda database of predicted targets. However, when compared with TargetScan predicted target genes, we observed only 6% overlap between significantly correlated and differentially regulated gene sets (Table S2). The intersection of all three data sets is the potential candidate target gene set, which represents 3% of the originally differentially regulated genes (Table S2). We applied this methodology to the following up-regulated miRNAs under LPS stimulation: miR-155, -297-5p, -302c, -10b, -495, -7a, -376a, -539, and -876-5p and the following down-regulated miRNAs under LPS stimulation: miR-124, -2132, -2122, -143, -219, -700, -689, -762, -1901, -466b-3p, -192, -383, -3474 and -1928. Using miR-155 as an example of this methodology to identify potential miRNA:mRNA interactome, we first identified 4231 genes that were strongly correlated with miR-155 expression and significantly differentially expressed under LPS stimulation (p<0.0001). We then compared the identified genes to the 1081 genes identified as potential miR-155 targets by targeScan/IPA database, and to the 3329 genes identified as predicted miR-155 targets by the miRanda database. The intersection of these three data sets netted a total of 112 potential miRNA-155:mRNA interactions, of which 16 were previously validated targets of miR-155 (Fig. 4B and Table 2).

**Table 2:** LPS differentially regulated genes correlated with miR-155 expression levels.

Symbol	Description	Correlation Factor	Log2 Fold Change	Power
**Up-regulated miR-155 correlated genes**				
Socs1	Suppressor of cytokine signaling 1	0.89	2.744	1.96E-08
Gpr85	G protein-coupled receptor 85	0.92	2.211	1.67E-07
Ell2	Elongation factor RNA polymerase II 2	0.93	2.094	6.35E-10
Fam102b	Family with sequence similarity 102, member B	0.93	1.843	2.05E-08
B3gnt5	UDP-GlcNAc:betaGal beta-1,3-N-acetylglucosaminyltransferase 5	0.95	1.721	1.74E-08
Fgl2	Fibrinogen-like protein 2	0.87	1.695	4.13E-07
Ets1	E26 avian leukemia oncogene 1, 5' domain	0.91	1.523	8.64E-08
Hivep2	Human immunodeficiency virus type I enhancer binding protein 2	0.95	1.476	5.03E-08
Mier3	Mesoderm induction early response 1, family member 3	0.95	1.403	4.90E-08
Cebpb	CCAAT/enhancer binding protein (C/EBP), beta	0.86	1.386	5.30E-07
Il13ra1	Interleukin 13 receptor, alpha 1	0.88	1.382	1.24E-07
Rapgef2	Rap guanine nucleotide exchange factor (GEF) 2	0.94	1.310	1.39E-07
Spred1	Sprouty protein with EVH-1 domain 1, related sequence	0.96	1.254	1.59E-07
S1pr1	Sphingosine-1-phosphate receptor 1	0.89	1.249	9.70E-08
Ikbke	Inhibitor of kappaB kinase epsilon	0.94	1.234	4.68E-07
Sgk3	Serum/glucocorticoid regulated kinase 3	0.92	1.035	5.15E-08
Samhd1	SAM domain and HD domain, 1	0.92	1.023	9.24E-08
Gdf6	Growth differentiation factor 6	0.85	0.975	4.59E-05
Morc3	Microrchidia 3	0.87	0.888	1.66E-06
Myo1d	Myosin ID	0.80	0.837	3.79E-06
Rictor	RPTOR independent companion of MTOR, complex 2	0.92	0.837	2.27E-07
Tle4	Transducin-like enhancer of split 4, homolog of Drosophila E(spl)	0.96	0.795	8.86E-07
Satb2	Special AT-rich sequence binding protein 2	0.74	0.790	6.62E-05
Atad2b	ATPase family, AAA domain containing 2B	0.93	0.787	1.19E-06
Hif1a	Hypoxia inducible factor 1, alpha subunit	0.96	0.760	1.10E-06
Cd47	CD47 antigen (Rh-related antigen, integrin-associated signal transducer)	0.93	0.750	7.77E-06
Vcpip1	Valosin containing protein (p97)/p47 complex interacting protein 1	0.92	0.706	1.26E-06
Asf1a	ASF1 anti-silencing function 1 homolog A (S. cerevisiae)	0.91	0.694	1.84E-06
Whsc1l1	Wolf-Hirschhorn syndrome candidate 1-like 1 (human)	0.91	0.663	4.19E-06
Cdk14	Cyclin-dependent kinase 14	0.91	0.660	6.07E-05
Syvn1	Synovial apoptosis inhibitor 1, synoviolin	0.82	0.641	3.91E-05
G3bp2	GTPase activating protein (SH3 domain) binding protein 2	0.91	0.623	4.05E-06
Xiap	X-linked inhibitor of apoptosis	0.96	0.611	5.02E-06
Lrrc59	Leucine rich repeat containing 59	0.85	0.595	1.41E-05
Kalrn	Kalirin, RhoGEF kinase	0.86	0.590	3.97E-06
Rab22a	RAB22A, member RAS oncogene family	0.86	0.578	6.59E-06
Med13l	Mediator complex subunit 13-like	0.73	0.542	6.31E-06
Pkn2	protein kinase N2	0.91	0.528	1.28E-05
Carhsp1	Calcium regulated heat stable protein 1	0.68	0.514	6.13E-05
Dmtf1	Cyclin D binding myb-like transcription factor 1	0.86	0.506	1.36E-05
Arl5b	ADP-ribosylation factor-like 5B	0.96	0.476	3.89E-05
Dennd1b	DENN/MADD domain containing 1B	0.81	0.476	1.57E-05
Kbtbd2	Kelch repeat and BTB (POZ) domain containing 2	0.94	0.451	5.61E-05
Ythdc2	YTH domain containing 2	0.89	0.426	9.64E-05
**Down-regulated miR-155 correlated genes**				
Fam105a	Family with sequence similarity 105, member A	-0.94	-3.370	7.86E-10
Inpp5d	Inositol polyphosphate-5-phosphatase D	-0.94	-2.482	3.53E-10
Arvcf	Armadillo repeat gene deleted in velo-cardio-facial syndrome	-0.92	-2.036	1.32E-07
Rapgef5	Rap guanine nucleotide exchange factor (GEF) 5	-0.92	-1.741	1.02E-08
Ssh2	Slingshot homolog 2 (Drosophila)	-0.88	-1.661	4.28E-07
Tspan14	Tetraspanin 14	-0.95	-1.537	2.80E-09
Myb	Myeloblastosis oncogene	-0.60	-1.434	2.77E-05
Reck	Reversion-inducing-cysteine-rich protein with kazal motifs	-0.94	-1.369	1.49E-07
Lat2	Linker for activation of T cells family, member 2	-0.91	-1.270	2.69E-07
Arhgap18	Rho GTPase activating protein 18	-0.92	-1.219	5.53E-08
Card11	Caspase recruitment domain family, member 11	-0.56	-1.165	5.49E-05
Mafb	v-maf musculoaponeurotic fibrosarcoma oncogene family, protein B (avian)	-0.78	-1.130	4.54E-08
Socs6	Suppressor of cytokine signaling 6	-0.66	-1.124	2.14E-07
Tmem144	Transmembrane protein 144	-0.83	-1.107	3.68E-06
Ddo	D-aspartate oxidase	-0.76	-1.102	3.64E-07
Entpd1	Ectonucleoside triphosphate diphosphohydrolase 1	-0.68	-1.077	1.05E-07
Rcbtb2	Regulator of chromosome condensation (RCC1) and BTB (POZ) domain containing protein 2	-0.79	-1.067	1.60E-06
Reps2	RALBP1 associated Eps domain containing protein 2	-0.63	-1.053	7.89E-07
Arhgap5	Rho GTPase activating protein 5	-0.77	-1.036	1.08E-06
Tcf7l2	Transcription factor 7-like 2, T-cell specific, HMG-box	-0.98	-0.9855	6.03E-06
Rreb1	Ras responsive element binding protein 1	-0.91	-0.986	1.43E-07
Agl	Amylo-1,6-glucosidase, 4-alpha-glucanotransferase	-0.93	-0.960	2.82E-07
Add3	Adducin 3 (gamma)	-0.80	-0.905	1.83E-07
Antxr2	Anthrax toxin receptor 2	-0.88	-0.903	1.19E-06
Satb1	Special AT-rich sequence binding protein 1	-0.79	-0.873	1.98E-06
Rnf166	Ring finger protein 166	-0.94	-0.866	5.91E-06
Enpp1	Ectonucleotide pyrophosphatase/phosphodiesterase 1	-0.75	-0.849	2.10E-06
Meis1	Meis homeobox 1	-0.88	-0.849	1.52E-05
Mphosph9	M-phase phosphoprotein 9	-0.84	-0.817	3.11E-06
Nfia	Nuclear factor I/A	-0.84	-0.809	2.78E-07
Lcorl	Ligand dependent nuclear receptor corepressor-like	-0.93	-0.791	4.67E-07
Ski	Ski sarcoma viral oncogene homolog (avian)	-0.90	-0.775	2.64E-05
Dcun1d4	DCN1, defective in cullin neddylation 1, domain containing 4 (S. cerevisiae)	-0.86	-0.758	5.91E-06
Syne1	Synaptic nuclear envelope 1	-0.88	-0.727	4.04E-06
Sort1	Sortilin 1	-0.89	-0.722	6.00E-06
Rell1	RELT-like 1	-0.72	-0.711	8.42E-06
Dixdc1	DIX domain containing 1	-0.73	-0.705	1.40E-05
Csf1r	Colony stimulating factor 1 receptor	-0.80	-0.692	8.23E-06
Haus3	HAUS augmin-like complex, subunit 3	-0.82	-0.680	1.44E-05
Gabra4	Gamma-aminobutyric acid (GABA) A receptor, subunit alpha 4	-0.83	-0.665	2.57E-06
Akap10	A kinase (PRKA) anchor protein 10	-0.91	-0.660	1.65E-05
Cep68	Centrosomal protein 68	-0.67	-0.659	8.79E-05
Rufy2	RUN and FYVE domain-containing 2	-0.96	-0.634	3.60E-06
Megf10	Multiple EGF-like-domains 10	-0.90	-0.611	1.75E-05
Tbck	TBC1 domain containing kinase	-0.91	-0.600	9.79E-06
Zc3h12b	Zinc finger CCCH-type containing 12B	-0.79	-0.597	4.85E-05
Fads1	Fatty acid desaturase 1	-0.91	-0.585	4.98E-06
Zmym2	Zinc finger, MYM-type 2	-0.87	-0.581	4.63E-05
Terf1	Telomeric repeat binding factor 1	-0.88	-0.574	2.03E-05
Tnpo1	Transportin 1	-0.86	-0.558	8.55E-05
Igsf11	Immunoglobulin superfamily, member 11	-0.67	-0.540	7.77E-06
Mbtd1	Mbt domain containing 1	-0.83	-0.533	5.42E-05
Trim2	Tripartite motif-containing 2	-0.76	-0.515	2.27E-05
Nudt4	Nudix (nucleoside diphosphate linked moiety X)-type motif 4	-0.78	-0.510	1.59E-05
Ogn	Osteoglycin	-0.72	-0.496	9.79E-05
Ergic1	Endoplasmic reticulum-golgi intermediate compartment (ERGIC) 1	-0.91	-0.480	2.18E-05
Ankrd12	Ankyrin repeat domain 12	-0.63	-0.479	1.91E-05
Csnk1g2	Casein kinase 1, gamma 2	-0.94	-0.479	1.96E-05
Pskh1	Protein serine kinase H1	-0.95	-0.467	2.98E-05
Slc39a10	Solute carrier family 39 (zinc transporter), member 10	-0.86	-0.448	3.47E-05
Clcn3	Chloride channel 3	-0.74	-0.442	7.51E-05
Grsf1	G-rich RNA sequence binding factor 1	-0.81	-0.422	4.48E-05
Rcor1	REST corepressor 1	-0.86	-0.417	3.78E-05
Asph	Aspartate-beta-hydroxylase	-0.85	-0.414	3.94E-05
Ikbip	IKBKB interacting protein	-0.86	-0.411	9.88E-05
Dclre1a	DNA cross-link repair 1A, PSO2 homolog (S. cerevisiae)	-0.90	-0.372	7.23E-05
Tram1	Translocating chain-associating membrane protein 1	-0.86	-0.365	7.87E-05
Fbxo22	F-box protein 22	-0.90	-0.351	7.44E-05

Interestingly, of the 112 potential miR-155 targets 68 were down-regulated and inversely correlated with the expression of miR-155, suggesting direct regulation by miR-155. The remaining 44 identified candidate targets were rather up-regulated and positively correlated with the expression of miR-155, including a number of known targets of miR-155, e.g. Suppressor of Cytokine Signaling-1 (SOCS1), hypoxic inducible factor 1-alpha (HIF1α) and CCAA/enhancer binding protein-β (CEBPβ) (Fig. 4B and Table 2). Most other predicted targets of specific miRNAs were evenly split (50/50) between those targets inversely correlated to their respective miRNA, as expected, versus those targets that were positively correlated with their respective miRNA (data not shown). This observation is consistent with previous miRNA:mRNA correlation observations [47].

**Figure 5:**
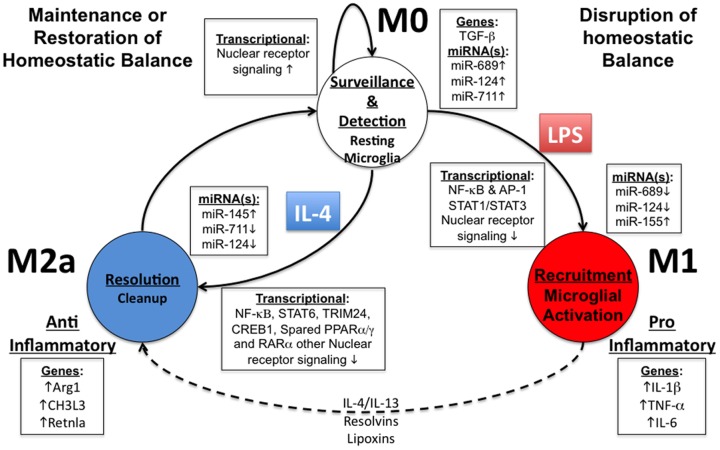
Proposed microglial phenotype transition model . At the M0 state (top), resting microglia function in a surveillance and detection mode, which appears to be regulated by various nuclear receptor pathways and select miRNAs: miR-124, miR-689 and miR-711. Upon detection of a danger or pattern molecule, the resting status is disrupted and transitions to the M1 state (right). The M1 phenotype is the “classic activation” status and prominently induces canonical M1 marker genes, e.g. IL-1β, TNF-α and IL-6. miR-124 and miR-689 are critical in initiation of the transition from the M0 to the M1 state. The M1 phenotype appears to be fully mediated by miR-155, which targets the STAT3 pathway for enabling the M1-phenotype. Later, through transition from M1 to M2 or through direct IL-4 stimulation (dashed line), microglia may enter the M2a status, characterized as an anti-inflammatory and resolution phenotype. As observed with in M1, down-regulation in miR-124 and miR-711 appears to be important for release from the M0-phenotype and transition to the M2 status. The M2a-phenotype appears to rely on induction of miR-145, which may regulate the ETS1 pathway. Lastly, IL-4 signaling is dependent on STAT6, TRIM24, and CREB1 along with select nuclear receptor signaling: PPARα/γ and RARα.

To determine how each miRNA potentially contributes in shaping the M1- or M2-phenotype of microglia, the second IPA enrichment analysis was performed using our lists of potential miRNA:mRNA interactions. miRNAs were selected based on a combination of p-values and an average expression level of greater than 50% as referenced to miR-709, the highest expressing miRNA in microglia in all three groups. M1-skewing of microglia yielded four up-regulated miRNAs: miR-155, -297b-5p, -10b, and -376a and six down-regulated miRNAs: miR-124, -2132, -2133, -700, -689, and -762. By combining the correlated miRNA:mRNA interactions from all of the miRNAs and the IPA transcriptional factor analysis, we observed over 153 transcriptional networks that are potentially altered though these miRNA:mRNA interactions (Fig 4C and Table S3). Interestingly, down-regulation of miR-689 and miR-124 appears to have more impact on these gene transcriptional networks than the up-regulation of miR-155 based on the –log (p-value) from the IPA enrichment analysis (Fig. 4C and Table S3). miR-689 potentially alters the transcriptional networks of many canonical pro-inflammatory pathways including NF-κB-RelA and NFATC2/3. Further, miR-124, a miRNA known to alter microglial phenotypes [27], was identified as potentially regulating ELK4, NCOA3, NFE2L2, and STAT1 transcriptional networks in this analysis. miR-155 potentially regulates three transcriptional networks, the most significant being STAT3, HOXA9, and CEBPβ (Fig 5C).

Collectively, we have observed a set of miRNAs for M1-skewed microglia and identified the potential transcriptional targets of miR-155. Further, our second IPA analysis identified down-regulation of both miR-689 and miR-124 as playing potential key roles in M1-activation of microglia. Overall, our data suggests that down-regulation of miRNAs, e.g. miR-689 and miR-124, may ‘release’ microglia from the M0 state, and up-regulation of miR-155 contribute to the establishment of the M1-skewing.

### Target analysis of key miRNAs identified in IL-4 stimulated murine primary microglia

We applied the same approach to identify key potential miRNA:mRNA interactions that regulate switching microglia from M0 to M2a phenotype. Overall, 20 different miRNAs that represent the most up- or down-regulated miRNAs under M2a skewing conditions were analyzed (Table S4). Interestingly, 1608 genes were significantly differentially regulated (p<0.0001) by IL-4 stimulated condition with similar overlap percentages as LPS stimulated condition: 13.27% overlap with the miRanda database, 6.90% overlap with IPA-provided database, and a combined overall overlap of 3.28% (Table S4). To identify critical potential miRNA:mRNA interactions that regulate switching from the M0 to the M2a phenotype we performed correlation analysis on the following up-regulated miRNAs: miR-449a, - 295, -145, -297-5p, and -214 and the following down-regulated miRNAs: miR-124, -154*, -2133, -384-5p, -2135, -3473, -326, -2132, -133, -383, -2861, -2138, -762, -1224 and -711. Overall, this process identified 724 potential targets, representing 66% of correlated genes under IL-4 stimulation of microglia that are potentially regulated by miRNA:mRNA interactions.

Using the same screening criteria as described above, we performed the second IPA enrichment analysis on four up-regulated miRNAs upon IL-4 stimulation: miR-145, -214, -297b-5p, and -449a and nine are down-regulated miRNAs: miR-711, -124, -2133, -2135, -2132, -2861, - 2138, -762, and -1224. Under M2a-skewing conditions we identified over 268 transcriptional networks that are potentially altered by miRNA:mRNA interactions. Interestingly, down-regulation of miR-711 or mir-124 are as significant as up-regulation of either miR-145 or miR-449a in establishing the M2a phenotype based on –log(p-value) score (Fig 4D and Table S5). miR-711 appears to alter the transcriptional networks of many canonical pro-inflammatory pathways including NF-κB-RelA, IRF1/2, SP1, IκB, and AP1 (Fig 4D and Table S5).

Collectively, we have identified a set of miRNAs for M2a-skewed microglia, which may play a critical role in the IL-4 response. Further, our second IPA analysis identified that down-regulation of miR-711 or miR-124 may play a key role in ‘releasing’ microglia from the M0 state, and up-regulation of miR-145 may contribute to establishment of the M2a state.

## Discussion

### miRNAs in M1 and M2a phenotypes

In the present study, we systematically identified key miRNAs and their potential role in regulating murine microglial M1 or M2-skewed activation. To the best of our knowledge this is the first genome-wide study combining both mRNA and miRNA expression profile of primary cultured murine microglia.

We identified mmu-miR-155 as the primary miRNA induced upon M1-skewing of microglia. Interestingly, miR-155 plays a key role in regulating the acute innate immune response in peripheral macrophages [24], [25], [48], [49]. In addition, in peripheral macrophages, miR-155 is strongly induced upon several TLR ligands, as well as bacterial or viral infection, oxidized LDL, TNF-α, and interferon-γ. (for review, see O’Neill *et al*., 2011[26]). Further, our finding corroborates Cardosa, A, *et al*.’s 2011 study that also demonstrated up-regulation of miR-155 upon LPS stimulation of primary cultured microglia and in the N9 microglial cell line [50]. Our study demonstrated that miRNA prominently altered in the M1-skewed microglia is limited to miR-155 and any other miRNAs, e.g. miR-10b and miR-297b-5p are only modestly altered (< 2-fold change). miR-155 is likely to be the key miRNA that mediates an acute pro-inflammatory state in primary microglia, demonstrating that miR-155 is an apparent therapeutic target for M1-skewed microglia-related neurologic disorders.

IL-4 stimulation is known to induce alternative activation in primary microglia [8]. We observed several miRNAs that are significantly up-regulated in IL-4 stimulated primary microglia, including miR-145 and mir-214. The association of miR-145 or miR-214 with the IL-4/STAT6 signaling pathway has never been reported in microglia. Interestingly both miR-145 and miR-214 are anti-oncogenic miRNAs [51]–[54]. Supporting a role for miR-145 in the IL-4/STAT6 signaling, Collison A. *et al.* have recently demonstrated that miR-145 inhibition is as effective as steroid treatment in animal models of allergic airway disease, a disease mediated by IL-4 induced Th2 skewing disorder [55]. miR-214 has no known roles in either microglial or macrophage biology. However, Jindra, PT *et al*. reported a role for miR-214 in T cell proliferation [56], suggesting a similar function as miR-145.

### Critical regulators determining microglial phenotypes

Since miRNAs are known to regulate entire ‘batteries’ of genes leading to phentotypic switches, we first sought to identify key regulatory networks and transcriptional pathways that are altered between different phenotypic states in microglia [57]. M1-skewed microglia demonstrated activation of many well-characterized pro-inflammatory transcriptional networks, including NF-κB, AP-1, STAT 1-4, and IRF 1,3,7, and 8 [58]–[60]. On the other hand, M2a-skewed microglia demonstrated a unique activation of pathways primarily though IL-4 signaling molecules including STAT6, CREB1 and IRF2 [61]–[63]. Further, we observed differential regulation of the JAK/STAT transcriptional networks: M1-activation suppresses the STAT 6 network, while M2a-activation suppresses STAT 1-4 networks (Fig 3A). Lastly, we observed profound suppression of the nuclear receptor family pathways under both M1 and M2-skewing conditions, suggesting the suppression of their activity to release microglia from the M0 state.

### JAK/STAT Signaling in Microglial Activation

We observed significant differences in the transcriptional regulation between LPS and IL-4 stimulation in microglia, particularly in the activation patterns of the JAK/STAT pathways. At the four-hour time point, STAT3 and STAT1 signaling dominate the active transcriptional network in LPS stimulated microglia. This is consistent with similar observations in peripheral macrophages stimulated with LPS that demonstrate marked reductions in NF-κB signaling after ∼2 hours and strong up regulation of STAT1/STAT3 signaling [59], [60]. The neuroinflammatory role of STAT3 in microglia has been suggested by multiple studies as inhibition of STAT3 mitigates LPS-induced activation of microglia [64], [65]. Further, to underscore the prominence of STAT3, Qin, H. *et al*. recently demonstrated that a conditional knockout of SOCS3, an endogenous STAT3 antagonist, increases the severity of EAE [66]. Our study shows that STAT3/STAT1 axis is one of the primary activating signal pathway in LPS-stimulated microglia at the 4 hr time point.

While STAT3 and STAT1 help establish the M1-skewing of microglia, M2a-skewed microglia are facilitated by STAT6 signaling pathway [61], [67]. Under IL-4 stimulation, microglia show strong activation of the STAT6 signaling pathway (Fig. 3A). Interestingly, we also observed strong down-regulation of STAT6 signaling by LPS stimulation, suggesting active repression of the STAT6 pathway may be necessary for M1-skewing of microglia. In accord, under IL-4 stimulation we observed significant inhibition of the STAT1/3 signaling pathways, suggesting repression of STAT1/3 pathway is equally necessary for M2-skewing of microglia. Taken together these data suggest cross-inhibition between the STAT1/3 pathways and the STAT6 pathway for efficient M2 or M1-skewing of microglia.

### Nuclear Receptor Alterations in Microglial Activation

Under either M1 or M2a-skewing of primary microglia activation we observed marked reductions in the transcriptional activity of many nuclear receptors relative to the M0 resting phenotype (Fig 3C) including: PPARα/γ/δ, Liver X receptor-alpha/beta (LXRα/β), glucocorticoid receptor (NR3C1), and the retinoic acid receptor-alpha (RARα). There is a growing body of literature linking innate inflammatory processes under M1-skewing conditions to reduced activation of the various nuclear receptors, e.g. PPARγ/δ, RXR and LXR systems [45], [68]–[73]. The necessity of reduced nuclear receptor activity in enabling the M1 phenotype is underscored by the ability of PPARγ/δ, RXR or LXR nuclear receptor agonists to blunt many of the pro-inflammatory mediators under LPS stimulation [69]–[71], [73]. Further, these nuclear receptor agonists have all demonstrated the ability to exert anti-inflammatory activity in peripheral macrophages and reduce macrophage-mediated disorders such as atherosclerosis [74]–[76]. In microglia, treatment with nuclear receptor agonists (e.g. PPARα/γ, RXR and LXR) has been shown to exert similar anti-inflammatory activity [77]–[79]. Recently, Achiron, A. *et al*. demonstrated dysregulation of the nuclear receptor family in human peripheral blood in pre-disease state of MS, suggesting this is a critical aspect in the progression of MS from pre-clinical to clinical states [45].

Based on the evidence linking nuclear receptor activation with anti-inflammatory activity, we had originally anticipated that M2a-skewing to up-regulate the activity of the nuclear receptor family. Surprisingly, many of the same nuclear receptors that were down regulated upon M1-skewing were also down regulated in M2a-skewing, albeit to a much lesser extent. However, we observed a critical difference between the M1-skewing and M2a-skewing phenotypes in the activity of the PPARγ and RARα transcriptional networks, which both demonstrated a greater than 3-fold difference in activity between the two phenotypes (Fig 4C). Interestingly, therapeutic application of the PPARγ agonists, pioglitazone, has been demonstrated to skew microglia from M1 to the M2 phenotype, reduce amyloid plaque burden, and improve cognitive deficits in the APP/PS1 mouse model of AD [80]. Thus, it is highly likely that phenotypic switch of microglia from M1 to M2-skewed activation is therapeutically relevant for neurologic disorders involving neuroinflammation.

### Inferring miRNA’s role in regulating M1 and M2 phenotype

We are intrigued that many of our identified miRNA:mRNA interactions, even for validated known target mRNAs of the miRNA, did not always follow the canonical repression paradigm (Table 2). That is, the target mRNA was up-regulated despite up-regulation of an miRNA that has been predicted to interact and repress said target. Conversely, we also observed down-regulation in target mRNA where interacting miRNAs were also correspondingly down regulated. Taken together, this suggests a much more nuanced and complex regulatory mechanism employed, which Shahab, S. *et al.* recently observed using a similar systems analytical approach in ovarian cancer model [47].

These studies suggest a more complex scheme of regulation beyond simple down regulation of a target. Indeed, the majority of miRNA regulation is at the post-transcriptional level, and inhibition of translation may up-regulate gene transcription to compensate the reduced expression of target protein by miRNA. Thus, identification of miRNA:miRNA interaction itself may be more relevant than focusing on positive or negative correlation of mRNA:miRNA to elucidate the potential miRNA:mRNA interactome.

Far less is known about the role miRNAs play in shaping the M2a phenotype in either peripheral macrophages or primary microglia. Upon M2a-skewing of primary microglia we observed up-regulation in two miRNAs: miR-145 and miR-214. miR-145 was recently identified as down-regulated in M2-skewed peripheral macrophages as compared to that of M1-skewed macrophages while this paper was being revised [81]. As was observed under LPS stimulation, some miRNAs are down regulated that appear to facilitate initial activation. Specifically, during M2a-skewing down-regulation of miR-711 and miR-124 may work in coordination to release microglia from the resting state. miR-711 potentially regulates targets associated with a number of pro-inflammatory pathways including AP1, IRF1/2, NF-κB1-RelA, SP3, Krupple-like factor-2 (KLF2) and PPARγ. In accord, PPARγ directly regulates alternative activation in peripheral macrophages [82], and KLF2 directly regulates the pro-inflammatory state in monocytes and facilitates the switch to alternative activation to M2 phenotype respectively [82]–[84], supporting the role of PPARγ and KLF2 on M2-skewing.

### Overall Summary

Based on our study the following microglial phenotype (state) transition diagram is proposed (Fig. 5). To establish the M1-skewed activation of microglia, reductions of both miR-689 and miR-124 release microglia from resting (M0) state, and facilitate canonical TLR signal pathways and NF-κB-RelA effector pathways, enabling the initial pro-inflammatory “recruitment” M1 phenotype. The swift up-regulation of miR-155 may drive the transition from TLR signal and NF-κB/RelA pathways to STAT1/STAT3 signaling pathways to complete the late-phase elements of the M1-skewed response. For M2-skewed activation, the down-regulation of miRNAs is critical in ‘releasing’ primary microglia from their M0 state, through down regulation of miR-711 and miR-124, and up-regulation of miR-145 may facilitate establishing the M2a-alternatively activated state. Further studies will be necessary for examining the specific miRNA:mRNA interactions, their roles in activation phenotypes of microglia, and their clinical implications.

## Supporting Information

Figure S1 **Microglial Culture Purity and Experimental Scheme.** (S1A), murine primary microglial isolated using the Percoll® density gradient methodology cultured after 5 days, as describe in section 2.0 materials and methods. Microglia were stained with immunoflorescent antibodies to identify microglia (CD11b, green), astrocytes (GFAP, red), and neurons (MAP2, blue), demonstrating a greater than 85% purity. (S1B), murine primary microglial isolated using the magnetic bead methodology cultured after 5 days, as describe in section 2.0 materials and methods. Flow cytometry assessment of purity by staining isolated primary microglia with anti-CD11b-APC immunoflorescent antibody, demonstrating a greater than 93% purity. (S1C), Experimental protocol scheme, Primary microglia were isolated from P0-P1 pups and cultured 5-7 days. 24-hours prior to stimulation media was changed to serum free media and at time (t = 0) were stimulated with LPS (100 ng/ml), IL-4 (20 ng/ml), or PBS for four hours. Cells were then lysed and total RNA was harvested for both miRNA and mRNA microarray analysis (n = 3 per condition), as well as for mRNA RT-PCR and miRNA LNA-based RT-PCR (n = 4 per condition).
(TIF)

Figure S2 **Comparison of Percoll vs. Magnetic Beads Isolation of mRNA and miRNA RT-PCR Summary.** (S2A), mRNA RT-PCR Delta-delta CT values of select genes (IL-6, IL-1β, TNF-α, NOS2 and Arg1) to compare Percoll density gradient (Open columns) versus Magnetic bead (hashed colimns) isolation methodology of primary murine micoglia. Both cohorts of microglia were cultured for 5-7 days and the stimulated for four hours with LPS (100 ng/ml and identified by red bars), IL-4 (20 ng/ml and identified by blue bars) or, PBS stimulated resting microglia (n = 3 or 4, ***  =  p<0.001 and *  =  p<0.05). (S2B), Comparison of Percoll density gradient versus Magnetic bead isolation methodology of primary murine micoglia of miRNA RT-PCR Delta-delta CT values of miR-155 (n = 3 or 4, NS  =  non-significant difference).
(TIF)

Figure S3 **mRNA Summary Systems Biology Analysis: Functional Analysis and Comparison**. Gene enrichment analysis of differentially regulated mRNA expression data identifying the Top 10 highly enriched “Biological Functions” up-regulated (S3A) and down-regulated (S3B) in M1-classically activated primary microglia. The highly enriched functions were identified from Ingenuity® Knowledge Base functional analysis and ranked based on -Log(p-value) score. (p < 0.05 corresponds to –Log(p-value) > 1.30). Gene enrichment analysis of differentially regulated mRNA expression data identifying the Top 10 highly enriched “Biological Functions” up-regulated (S3C) and down-regulated (S3D) in M2a-alternatively activated primary microglia. The highly enriched functions were identified from Ingenuity® Knowledge Base functional analysis and ranked based on -Log(p-value) score. (p<0.05 corresponds to –Log(p-value) >1.30). (S3E) Representative detailed analysis of the “Inflammatory-Immune Response Function” by Venn-Diagram analysis demonstrates low (11.3%) commonality in enriched genes comprising the “Inflammatory-Immune Response Function” despite the same function being mapped in both M1-skewing and M2a-skewing phenotype.
(TIF)

Table S1 **Primer list for RT-PCR study.**
(XLSX)

Table S2 **LPS miRNA Correlation Analysis Summary.**
(XLSX)

Table S3 **LPS miRNA Targeting Systems Analysis Summary.**
(XLSX)

Table S4 **IL-4 miRNA Correlation Analysis Summary.**
(XLSX)

Table S5 **IL-4 miRNA Targeting Systems Analysis Summary.**
(XLSX)

Materials and Methods S1 **Supplementary Material and Methods.**
(DOCX)
