# Supplementary figures and images for: Integrated Expression Profiles of mRNA and miRNA in Polarized Primary Murine Microglia
Source: PLoS One. 2013 Nov 11;8(11):e79416. doi: 10.1371/journal.pone.0079416 (PMC3823621; doi:10.1371/journal.pone.0079416)

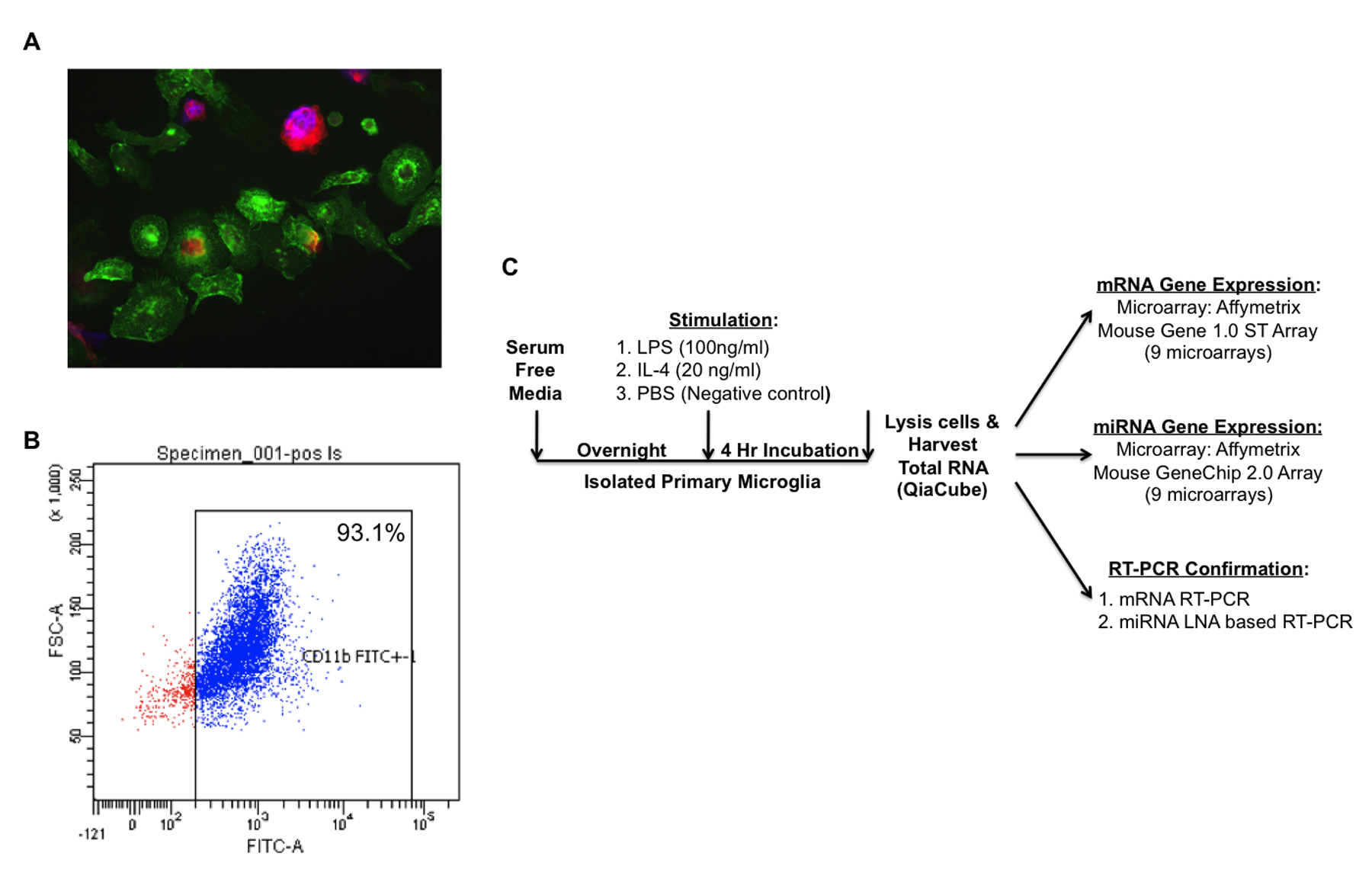

Supplement: Figure S1 — Microglial Culture Purity and Experimental Scheme. (S1A), murine primary microglial isolated using the Percoll® density gradient methodology cultured after 5 days, as describe in section 2.0 materials and methods. Microglia were stained with immunoflorescent antibodies to identify microglia (CD11b, green), astrocytes (GFAP, red), and neurons (MAP2, blue), demonstrating a greater than 85% purity. (S1B), murine primary microglial isolated using the magnetic bead methodology cultured after 5 days, as describe in section 2.0 materials and methods. Flow cytometry assessment of purity by staining isolated primary microglia with anti-CD11b-APC immunoflorescent antibody, demonstrating a greater than 93% purity. (S1C), Experimental protocol scheme, Primary microglia were isolated from P0-P1 pups and cultured 5-7 days. 24-hours prior to stimulation media was changed to serum free media and at time (t = 0) were stimulated with LPS (100 ng/ml), IL-4 (20 ng/ml), or PBS for four hours. Cells were then lysed and total RNA was harvested for both miRNA and mRNA microarray analysis (n = 3 per condition), as well as for mRNA RT-PCR and miRNA LNA-based RT-PCR (n = 4 per condition). (TIF) [file pone.0079416.s001.tif]

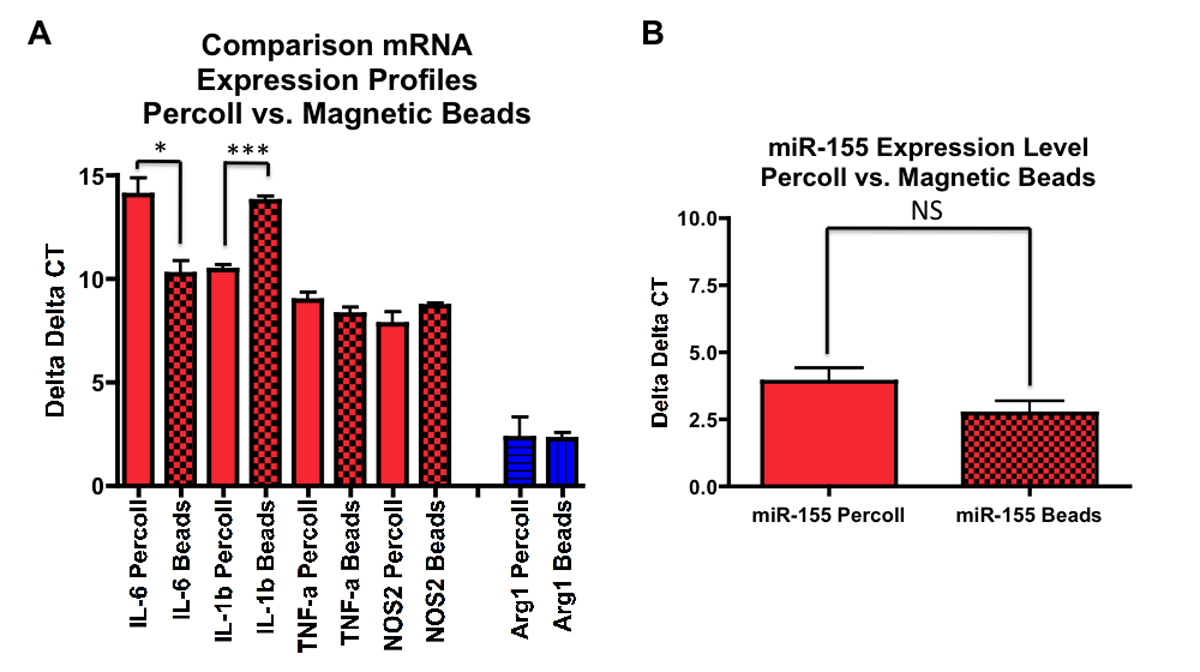

Supplement: Figure S2 — Comparison of Percoll vs. Magnetic Beads Isolation of mRNA and miRNA RT-PCR Summary. (S2A), mRNA RT-PCR Delta-delta CT values of select genes (IL-6, IL-1β, TNF-α, NOS2 and Arg1) to compare Percoll density gradient (Open columns) versus Magnetic bead (hashed colimns) isolation methodology of primary murine micoglia. Both cohorts of microglia were cultured for 5-7 days and the stimulated for four hours with LPS (100 ng/ml and identified by red bars), IL-4 (20 ng/ml and identified by blue bars) or, PBS stimulated resting microglia (n = 3 or 4, *** = p<0.001 and * = p<0.05). (S2B), Comparison of Percoll density gradient versus Magnetic bead isolation methodology of primary murine micoglia of miRNA RT-PCR Delta-delta CT values of miR-155 (n = 3 or 4, NS = non-significant difference). (TIF) [file pone.0079416.s002.tif]

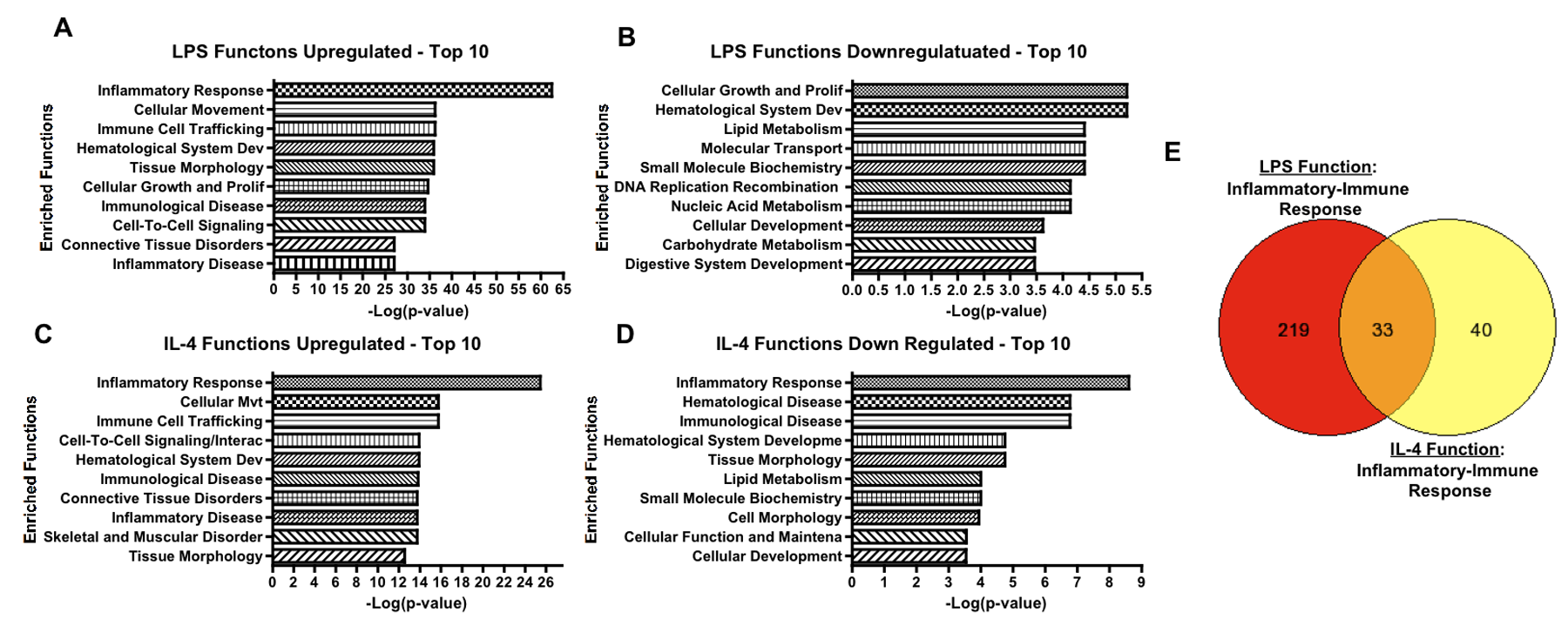

Supplement: Figure S3 — mRNA Summary Systems Biology Analysis: Functional Analysis and Comparison. Gene enrichment analysis of differentially regulated mRNA expression data identifying the Top 10 highly enriched “Biological Functions” up-regulated (S3A) and down-regulated (S3B) in M1-classically activated primary microglia. The highly enriched functions were identified from Ingenuity® Knowledge Base functional analysis and ranked based on -Log(p-value) score. (p < 0.05 corresponds to –Log(p-value) > 1.30). Gene enrichment analysis of differentially regulated mRNA expression data identifying the Top 10 highly enriched “Biological Functions” up-regulated (S3C) and down-regulated (S3D) in M2a-alternatively activated primary microglia. The highly enriched functions were identified from Ingenuity® Knowledge Base functional analysis and ranked based on -Log(p-value) score. (p<0.05 corresponds to –Log(p-value) >1.30). (S3E) Representative detailed analysis of the “Inflammatory-Immune Response Function” by Venn-Diagram analysis demonstrates low (11.3%) commonality in enriched genes comprising the “Inflammatory-Immune Response Function” despite the same function being mapped in both M1-skewing and M2a-skewing phenotype. (TIF) [file pone.0079416.s003.tif]
