# Supplementary material for: Integrated Expression Profiles of mRNA and miRNA in Polarized Primary Murine Microglia
Source: PLoS One. 2013 Nov 11;8(11):e79416. doi: 10.1371/journal.pone.0079416 (PMC3823621; doi:10.1371/journal.pone.0079416)
Supplement: Materials and Methods S1 — Supplementary Material and Methods. (DOCX) [file pone.0079416.s009.docx]

**Materials and Methods S1**

Microarray data analysis: To identify canonical pathways and functions previously ascribed to the significantly differentially expressed mRNAs and miRNAs were uploaded and analyzed using Ingenuity® Pathway Analysis (IPA) tool, a commercially available web-delivered bioinformatics tool, December 18, 2011 release (Ingenuity Systems, Redwood City, CA, USA – http://www.ingenuity.com) [[29](#_ENREF_29),[30](#_ENREF_30)]. Ingenuity functional analysis, canonical pathway analysis, and transcriptional factor analysis were identified by performing Ingenuity Pathway Analysis (IPA) core analysis of log2 fold change for LPS or IL-4 stimulated versus un-stimulated primary microglia microarray data sets.

The **Functional Analysis** of the observed LPS or IL-4 data sets identified the biological functions that were most significant to the data set. Molecules from the dataset that were associated biological functions in the Ingenuity Knowledge Base were considered for the analysis. Right-tailed Fisher’s exact test was used to calculate a p-value determining the probability that each biological function assigned to the data set is due to chance alone. Further, A regulation Z-score is calculated according to the fit of our significantly differentially regulated genes, where a Z-score > 2 or a Z-score <2 indicates a 99% confidence of not being generated by random chance alone for up-regulated functions or down regulated functions respectively.

**Canonical pathway analysis** identified the pathways from the IPA library of canonical pathways that were most significant enriched in either LPS or IL-4 stimulated primary microglia. Molecules from either data set that were associated with a canonical pathway in the Ingenuity Knowledge Base were considered for the analysis. The significance of the association between the data set and the pathway was measured in 2 ways: 1) a ratio of the number of molecules from the data set that map to the pathway divided by the total number of molecules that map to the canonical pathways is displayed. 2) Fisher’s exact test was used to calculate a p-value determining the probability that the association between the genes in the observed values and the canonical pathway is explained by chance alone.

**Transcriptional factor analysis** identifies activated or inhibited transcription factors based on the enrichment and direction of change of known target genes in the Ingenuity Knowledge Base and their association to transcription factors that were most significant enriched in either LPS or IL-4 stimulated primary microglia. The regulation Z-score is used to identify transcriptional regulators and infer their activation state. The significance of the association between the data set and the transcription factors was measured Fisher’s exact test was used to calculate an overlap p-value determining the probability that the association between the genes in the observed values and the genes known to be regulated by a specific transcription factor is explained by chance alone.
